# Robust Assembly Assistance Using Informed Tree Search with Markov Chains

**DOI:** 10.3390/s22020495

**Published:** 2022-01-10

**Authors:** Arpad Gellert, Radu Sorostinean, Bogdan-Constantin Pirvu

**Affiliations:** 1 Computer Science and Electrical Engineering Department, Lucian Blaga University of Sibiu, 550025 Sibiu, Romania; radu.sorostinean@gmail.com; 2 School of Electronics and Computer Science, University of Southampton, Southampton SO17 1BJ, UK; 3 Industrial Engineering and Management Department, Lucian Blaga University of Sibiu, 550025 Sibiu, Romania; bogdan.pirvu@ulbsibiu.ro

**Keywords:** assembly assistance systems, training stations, smart manufacturing, Industry 4.0, digital transformation, informed tree search, A* algorithm, Markov chains, prediction, artificial intelligence

## Abstract

Manual work accounts for one of the largest workgroups in the European manufacturing sector, and improving the training capacity, quality, and speed brings significant competitive benefits to companies. In this context, this paper presents an informed tree search on top of a Markov chain that suggests possible next assembly steps as a key component of an innovative assembly training station for manual operations. The goal of the next step suggestions is to provide support to inexperienced workers or to assist experienced workers by providing choices for the next assembly step in an automated manner without the involvement of a human trainer on site. Data stemming from 179 experiment participants, 111 factory workers, and 68 students, were used to evaluate different prediction methods. From our analysis, Markov chains fail in new scenarios and, therefore, by using an informed tree search to predict the possible next assembly step in such situations, the prediction capability of the hybrid algorithm increases significantly while providing robust solutions to unseen scenarios. The proposed method proved to be the most efficient for next assembly step prediction among all the evaluated predictors and, thus, the most suitable method for an adaptive assembly support system such as for manual operations in industry.

## 1. Introduction

To compete successfully in the global market, in recent years, factories have turned their attention to optimize all tasks, including the ones performed by humans, leveraging the progress in information technology with the deployment of artificial intelligence [1,2] and machine learning [3] in various application areas throughout the product life cycle. Industry 4.0 [4] is the coined term used to describe this optimization involving interconnection and collaboration among the factory’s interactants (human and synthetic) towards a human–automation symbiosis.

Nowadays, the adoption process in Industry 4.0 mainly focuses on assisting humans with different technologies in order to ease their tasks and improve productivity, as envisioned in the Operator 4.0 concept [5]. This is because full automation is costly and not effective in all areas due to dexterity, flexibility, and complexity requirements. Thus, assistance systems are required to facilitate interconnection and collaboration between humans and synthetic systems, especially in the case of manual work. Recent assembly assistance systems target multi-modal interaction with the user and are capable of providing adaptive instructions (i.e., type, content, sequence) tailored for different user categories (e.g., experience, age, gender) that take into account the user’s basic emotion and/or mental state. Smart anomaly detection and prediction can significantly reduce unexpected production interruptions [6]. Studies such as in [7] indicate that the impact of assembly assistance systems increases in the case of complex products and untrained persons.

This paper proposes a novel way of tackling assembly assistance prediction problems by improving the accuracy of commonly used Markov chain methods. One of the many problems involved in assembly assistance systems is the spread of the data inside multiple bins with a low amount of data in each bin. This confuses the Markov chain, causing low probabilities of each move due to the spread of the data; it can also cause a low prediction rate during testing when the algorithm meets new scenarios for which it was not trained.

The novelty of the proposed technique is the hybrid approach that combines an informed tree search with a Markov model, as well as the usage of this hybrid method in assembly assistance systems for next manufacturing step prediction. The scope of this paper is to address the problem described above by using an informed tree search on top of the Markov chain prediction to provide a plausible move for the next step when the Markov chain fails its prediction due to insufficient training data or meeting a new scenario. The predictions of the next move will always follow Markovian probability, and when all the probabilities are 0 or the Markov chain does not return any prediction, the following move will be chosen out of the plausible moves ranked by the heuristic function. The main goals are to identify, produce, and test different techniques that improve the prediction generated by Markov chains in scenarios that were not met during the training phase. This paper also aims to produce valid algorithms that can be used in an assembly assistance system, to evaluate the algorithm on the provided dataset and to prove its general usage, and to define any further work that can be done to improve the algorithm.

The rest of this paper is organized as follows. Section 2 presents the related work, Section 3 describes the proposed prediction method, Section 4 discusses the experimental results, and Section 5 concludes the paper.

## 2. Related Work

A summary of the most recent articles in the field of assistance systems, both technology- and impact-wise, is presented below. A comprehensive literature review (121 papers) on worker assistance systems used in the shop floor (i.e., in-situ), including the state-of-the-art, possible future directions, and the limitations of these systems, can be found in [8]. It reveals that although in its infancy, the research field of worker assistance has gained more attention with more industrial adoption, needing to develop more assistance applications that can help operators at their workstations on the shop floor, with a focus on user interface design and biomedical engineering as well. Methodologies for selecting the most suitable assistance systems for various user groups are missing, as well as a structured suitability evaluation of worker assistance in manufacturing. A further literature review (234 articles) has been targeted by Miqueo et al. [9], focusing on the manual assembly process and particularly targeting mass customization demand while considering the new technologies within Industry 4.0, lean aspects, and the impact on operators as well. The article concludes that “product clustering, modularization, delayed product differentiation, mixed-model assembly, and reconfigurable assembly systems” are essential for competitive assembly operation in mass customization and that augmented and virtual reality technologies are the key enabling technologies for supporting the operator. Worker assistance systems are analyzed in [10] from a manufacturing process perspective, covering more than 200 applications. The study revealed that most applications are for assembly processes, followed by cutting, welding, disassembly, and coating from the liquid state manufacturing process.

In [11], Tocu et al. developed and evaluated a virtual reality assembly process simulator. The experiments were performed on two groups of trainees. One group participated in virtual reality training before real training. The other group attended only the real training session. The results have shown that the additional training in a virtual reality environment significantly improved the efficiency of the workers in assembling a customizable modular tablet. Due to the training stations, human trainers can be more effectively used. A virtual reality training system for preparing operators to execute simple and infrequent maintenance tasks for Kuka robots is introduced in [12]. The solution relies on the HTC Vive Pro ecosystem for the hardware, while for the virtual reality application, the Unity game engine and SteamVR framework were used. The performance of the virtual reality training system was evaluated in an experiment with 18 persons, indicating a training performance similar to a standard one, although executed faster in virtual reality. Nevertheless, a higher cognitive load and greater frustration were observed in the case of the virtual reality approach.

Zheng et al. presented in [13] an augmented-reality-based assistant system for aircraft cable assembly with a convolutional neural network for deep vision that provides rapid guidance, reduces errors, and mitigates the dependency on hard copy documents. Another deep learning visual assistant for assembly tasks in production, recognizing real-time tools and worker assembly actions to reduce rework and waste, is presented in [1]. The proposed solution relies on a generic description language that was developed to characterize the actions within an assembly process. Another augmented-reality-based system for the high-end footwear industry using the Microsoft HoloLens is detailed in [14]. The prototype’s use-case is for the offline training of inexperienced workers for leather footwear assembly. The preliminary evaluation involved two apprentices over three days of testing and indicated benefits compared to the standard approach, such as the reduced number of interventions from experts, more adaptive user content, and less pressure during training. The main drawbacks were related to the low usability of the Microsoft HoloLens when using them for prolonged periods and that users were unaware of the mistakes made during training. This could be potentially improved with HoloLens 2. In [15], Vanneste et al. describe an augmented reality system as well as an ongoing experiment about the effects (i.e., time, errors, perceived complexity, required assistance, well-being variables) of exposing users to different instruction complexities for performing five main tasks. The experiment targeted overall 100 participants; no conclusions are yet formulated because only data from 23 persons were gathered. In [16], augmented-reality-based systems (HoloLens and smartphone) were evaluated in an experiment involving 12 persons to identify their training effectiveness compared with paper-based instructions. Fewer errors and improved usability were observed in comparison with paper-based instructions. No statistically relevant differences were identified from task completion time and overall workload perspective when comparing them. An extensive literature review (52 articles) of current extended-reality technologies (virtual reality, augmented reality, and mixed reality), their application for training in manufacturing, and the benefits and limitations can be found in [17].

A cognitive assistance system as a flexible and adaptive assistive assembly solution (i.e., deskbound and deskless) that is suitable for a dynamic environment (e.g., a rework area) is detailed in [18]. The system supports users regardless of their experience, providing work instructions as gif-animations as well as process-related data. Moreover, the user can interact with the system by having virtual buttons available. A limitation of the presented solution consists of the fact that it is not suitable for a product with more recipes to assemble or disassemble the product. The impact of task complexity and informational assistance systems is focused on in [19]. Here, the main conclusion after analyzing the mental workload via three physiological indicators (electrocardiography, heart rate variability, eye movement assessment) is that content and structure of the instructions are most important, while the type of assistance system or medium (i.e., paper, tablet, augmented reality device display) is irrelevant (given that the solution used has appropriate usability). A strategy for cognitive automation of Operator 4.0 can be found in [20]. 

An approach for designing better instructions, considering cognition, is tackled in [21], where a novel framework for developing such systems is described. Rupp and Müller presented in [22] a general approach and first implementation of a modular event-driven architecture assistance system to support operators at their workplaces in prototype and pre-series production. The instructions are generated directly from higher planning systems, while the assembly visualization is done in 3D, relying on the drawing database in conjunction with data from the product data management system. Approaches regarding the automation of content creation for assembly instructions are described in [23].

In [24], Bertram et al. evaluated 10 assistance systems for assembly operations from 6 aspects relevant for intelligent working stations and concluded that none of the 10 systems fully covers the automated generation of work plans, flexible integration in production, or autonomous learning ability. The benefits of gamification (e.g., faster training, less errors) versus a non-gamified approach were revealed in a study involving 50 participants by Palmas et al. [25]. In [26], several heuristic-based approaches were used in order to optimize picking orders in fulfillment warehouses. In particular, novel variants of column generation, genetic algorithms, and artificial neural networks that use heuristic-based optimization were applied to decrease the search domain and provide an optimal solution by decreasing the makespan in fulfillment centers. 

Next assembly step modeling, as a component of an adaptive assembly assistance system, has been analyzed in several papers. In [27], two-level context-based prediction has been proposed for the assembly process modeling of a customizable modular tablet. In [28,29], Markov chains have been involved in predicting the next assembly step. Markov chains have also been used in computational biology [30], web access mining [31], image processing [32,33], and energy management systems [34]. Markov models are very efficient for prediction in situations met before but inefficient in new situations. The Prediction by Partial Matching with Neighboring (PPMN) algorithm combines different order Markov models and has been used in [35]. In [36], a Long Short-Term Memory (LSTM) was applied. LSTM is usable in unmet situations, with good coverage, but its drawback is the lower accuracy. Until the current research, the Gradient Boosted Decision Tree (GBDT), evaluated in [37], has proved to be the most performant in predicting and suggesting the next assembly steps. In [37], there are several advantages to using GBDT, mainly maintaining a high prediction rate of 100% and a coverage of 65%. The disadvantage of using GBDT comes from the increased complexity of the algorithm, resulting in high implementation times and high data levels used as it presents an increased risk of overfitting. Another approach used in [38] is to compare different methods for assembly assistance and modeling based on the time cycle between the moves of the human operator. While this approach was considered, the time between assembly moves was not present in the data collected from the experiment [5].

While the vast majority of the literature focuses on achieving the highest prediction accuracy in next-step prediction, this paper approaches the problem using an informed tree search, such as in [39], in order to increase the prediction rate to 100%, thus covering all the possible scenarios. While a naïve heuristic predictor for the shortest path will not yield good accuracy due to all steps being the same cost, this will help the overall system by successfully dealing with unmet scenarios in the prediction phase, especially when working with a low amount of data. 

Tree search was analyzed in this paper because it offers the highest robustness in the assembly process, being able to deal with wrong assemblies by following the heuristic score when a prediction cannot be made by the Markov chain.

## 3. Next Assembly Step Prediction by Tree Search

To approach the prediction, several methods were considered. In this paper, a novel hybrid predictor based on heuristic tree search with a Markov model is explored. The approach focuses on achieving full coverage and being robust to recommend moves from any given start state. The algorithm is built by combining a tree search with an A* heuristic based on the Markov prediction implementation described in [4]. Section 3.1 covers the problem context, goals, and data analysis. In Section 3.2, the algorithm design and implementation are shown.

### 3.1. Assembly Assistance System

**Figure 1:**
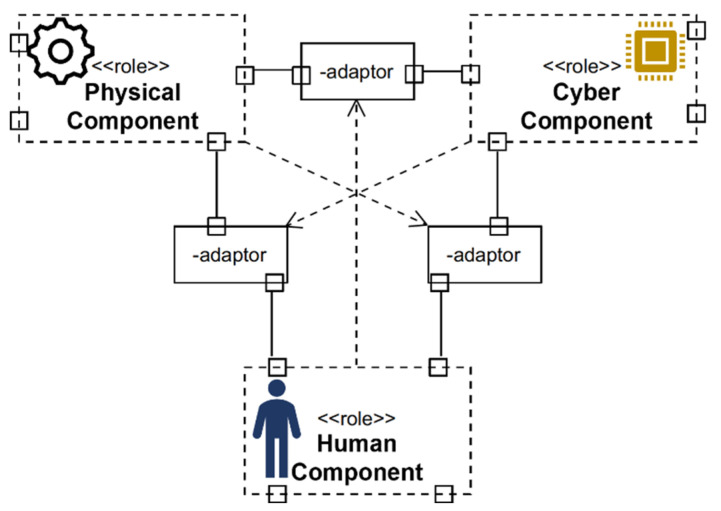
UML representation of the anthropocentric cyber–physical system reference model.

The assembly assistance system is a flexible training prototype for manual operations that allows the deployment and evaluation of adaptive training effects in the case of inexperienced operators without the support of an expert human trainer. Conceptually, it was designed to go beyond the classical approach of a cyber–physical system, in which a human–machine interface is encapsulated within a device, by following the anthropocentric cyber–physical system reference model approach [40] (see Figure 1). In anthropocentric cyber–physical systems, the social dimension is an integral part within its infinitesimal model and is essential for continuous adaptation between the physical, cyber, and human components.

**Figure 2:**
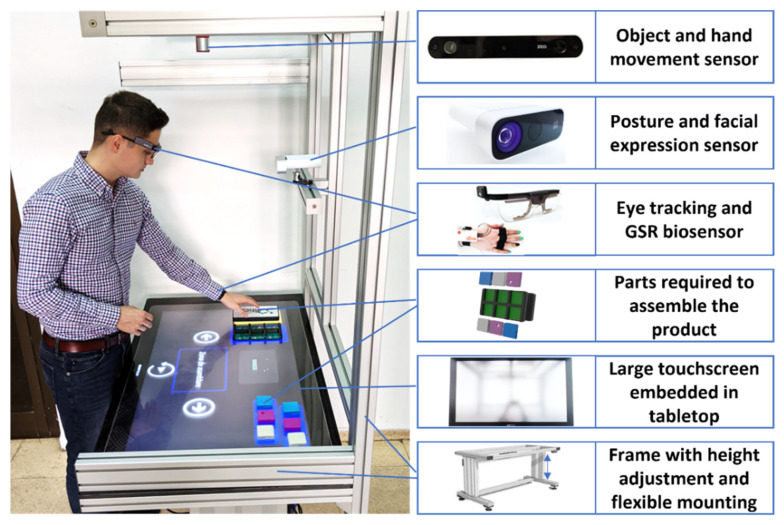
Assembly assistance prototype for manual operations.

From a hardware perspective, the prototype (Figure 2) is a flexible testbed due to its mechanical structure, allowing a configuration with all the required devices (e.g., touchscreen, cameras, sensors) in different training scenarios for the assembly of various small and medium products. 

The assembly assistance system is composed out of five main components. The first component is the aluminum profile frame designed for the easy positioning and installation of the required devices. Electrical actuators are embedded in the frame to vertically adjust the tabletop. The second component is a Sensytouch ST43 SLIM large touch screen. It is used for displaying instructions and providing the hardware for running the training application (see Table 1 for its specification). The third component is a set of biosensors, such as Tobii Pro Glasses 2 and Shimmer GSR, which are worn by the user during the training. If required, additional sensors, such as for electroencephalograms, electromyography, and heart rate, can be added. The fourth component is a front-facing Microsoft Azure Kinect sensor for posture and facial expression estimation. Finally, the fifth component is the Zed 3D camera, positioned above the user and oriented towards the assembly table for tracking hands and the relevant components for the assembly training.

From a software application perspective, the prototype offers flexibility because the training scenario (e.g., time pressure, audio and/or video guidance) and the instructions can be adapted (e.g., more/fewer detailed verbal instructions, more/less detailed video animations) based on data from the sensors (front-facing camera) or biosensors worn by the user (e.g., eye tracking, galvanic skin response, electromyography, heart rate). The assembly assistance system has micro-services implemented to improve the user experience. All the micro-services communicate via Google’s Remote Procedure Call framework. Additionally, each micro-service has its own Health Check and Service Discovery mechanism. These mechanisms allow the selection of the services that will collaborate at any given time. Below are the microservices that impact the predictor or user assembly behavior:

- Height adjustment of the tabletop: the adjustment can be made manually by pressing the physical buttons on the station or automatically from the software.
- Depth camera streaming: this service allows the clients to control the depth camera as if they were connected to it. It exposes all the camera capabilities (e.g., RGB, depth, point cloud).
- Object detection: this service detects the position of objects in each image.
- Object position: this service combines object detection and depth camera microservices to establish the 3D position of objects relative to the camera. Additionally, it detects if the objects have been inserted correctly in their slots. In case of a wrong step, it will prompt the user to undo the action. This feature acts as a safeguard for the prediction service since it cannot detect incorrect assemblies (nor should this be its responsibility).
- Emotion detection based on face mimics: this service detects the emotion of the user based on a picture with his face.
- Human characteristics collector: this service collects human characteristics such as age and gender. The mood can be identified with the aid of the “emotion detection based on face mimics” microservice. These characteristics aid in the prediction process due to factors or preferences representative of a segment of the population.
- Predictor: this service receives information collected from the other services through an aggregator, and, based on its various algorithms, it should return the next recommended assembly step. Its role is to guide the trainees during their training stage and, optionally, to assist experienced workers by providing choices for the next assembly step.

**Figure 3:**
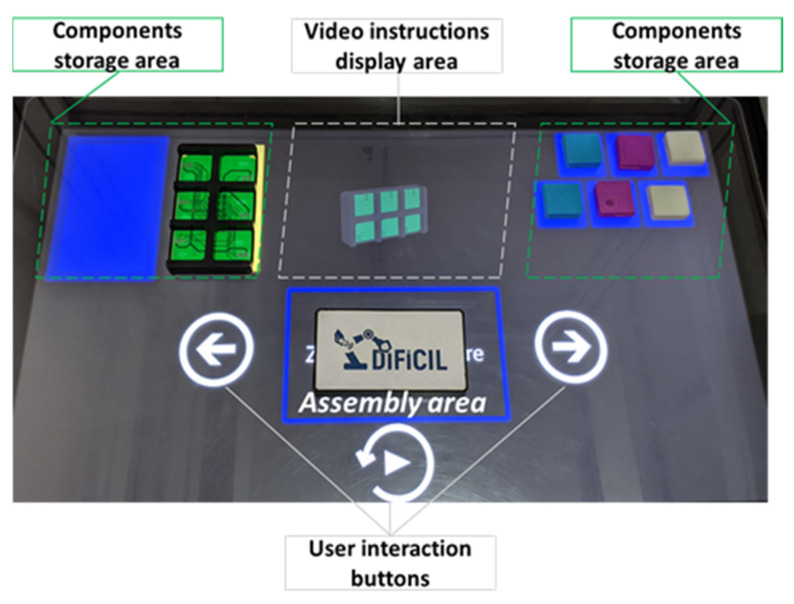
User interface of the assembly assistance prototype.

Before the training starts, the product’s components are placed disassembled in the designated areas (i.e., upper left side and upper right side) on the tabletop, as in Figure 3. The large touchscreen displays where the subcomponent’s initial location should be and provides video animations and user interaction buttons (i.e., play/pause, backward and forward), which enable the control of the training scenario. 

**Figure 4:**
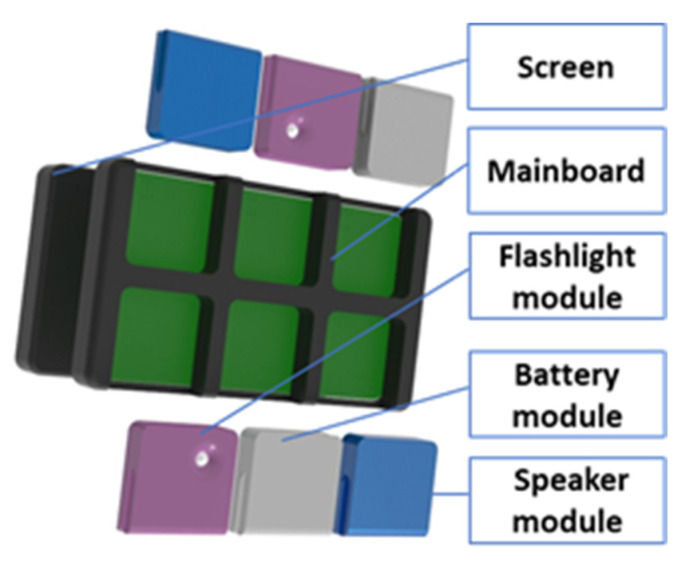
Example of a configuration for the modular, customizable product.

The product used in our evaluation is a customizable modular tablet (Figure 4) that can be configured with one screen, one mainboard, and up to six modules of three types (i.e., flashlight, battery, speaker). The assembly process can start with any of the components mentioned before, and it is not restricted to a single assembly recipe. More details about the prototype’s concept and its subsystems and feature developments are detailed in [41,42,43].

Using the assembly system described above, data were collected from 68 participants (second-year BSc students) and 111 manufacturing workers. The data was processed in order to describe the assembly process via a 7-bit representation, where the screen is the least significant bit and the bottom-right corner module represents the most significant bit. Each bit represents a slot on the mainboard, its value being 1 if the corresponding component is mounted correctly.

The data were processed following the same scenario as the one outlined in [29] to train the Markov model (described in that study). Mainly, quantitative and qualitative data were collected from each participant. The qualitative data were obtained through a questionnaire containing general questions about height, age, gender, dominant hand, highest level of education, and if the participants were eyeglass wearers. Some other questions were for self-evaluation: “Were you hungry during the experiment?”, “Do you have any prior experience in product assembly?”, “What was your stress level before the experiment?”, “How would you describe the state you found yourself in during the experiment (at the beginning, during, and at the end of the experiment)”, “Are you under the influence of any drugs that might influence your level of concentration?”, and ”How would you describe the sleep quality of the previous night?” From the collected qualitative data, we used height (tall or small), sleep quality (good or bad), gender (male or female), and whether the participant was wearing glasses (true or false). The quantitative data was the actual assembly of the tablet coded in bits. The mainboard was considered the reference for the other components. If a component was assembled on the mainboard in its correct slot, it was marked with “1”. If no component was assembled in a slot or the component was in the wrong slot, it was marked with “0”. With seven slots on the mainboard, there is a 7-bit code for the assembly state of the tablet. The assembly process of the tablet is represented as a sequence of assembly state codes. The quantitative data were used together with the qualitative data in training the algorithms. To mitigate the bias generated by the factory workers, who tend to assemble the tablet in sequential order compared to the students, the data was randomly mixed between the workers and the students. We reserved 75% of the data for training, and we used the remaining 25% for testing.

### 3.2. Algorithm Design

Using the Markov implementation described in [28], a novel hybrid approach was designed by adding A* informed tree search on top of the Markov predictor. We use the notations presented in Table 2.

The approach utilizes the predictions produced by the Markov chain at each prediction step in order to assign a score to each node under the parent state based on its probability of being the next move. A Markov chain can be defined as follows:

(1) $${P[s}_{t}|s_{t-1},\ldots {,s}_{1}]={P[s}_{t}|s_{t-1},\ldots {,s}_{t-R}]$$

A* is a graph traversal algorithm developed in 1968. The algorithm is used in several fields in computer science to solve the shortest path problem due to its completeness, optimality, and efficiency. The tree search is done using a heuristic function that is an estimate of the distance left to the goal. The term optimality is used to describe algorithms that, when used in tree search problems, are guaranteed to always return the best possible solution. The second term, completeness, is used to describe that if a solution to the problem exists, the given algorithm will always find it. The pseudocode for the implementation of the A* algorithm (Algorithm 1) can be found below.
**Algorithm 1.** A* algorithmqueue = [root] goal = goal_node while (queue not empty && queue[0] not goal){  current_node = queue.pop()  children = current_node.generate_children()  queue.add(children)  queue.sort(by = path_cost + heuristic) } if (queue[0] is goal){   return optimal_path(queue[0]) } else {   return “failure” } 

An admissible heuristic that can predict the exact distance from the current state q to the final state at each step was designed. The heuristic is

(2) H(q)=FSS(q)−D(q)−P(q)

where FSS(q) is the final state score, D(q) is the depth of the current state q, and P(q) is the probability of state q being the next state.

Since, at each move, the probabilities change, FSS is calculated based on the probabilities given by the Markov chain when predicting the next move from the parent node. Therefore, FSS can be calculated as follows:

(3) $$\mathrm{FSS}(q)=\mathrm{SD}(q)+\sum_{k=1}^{n}P(k)$$

where SD is the optimal solution depth. The final state score can be calculated in the context given in [29], where a tablet is assembled in 7 steps, as the optimal solution depth is known.

**Figure 5:**
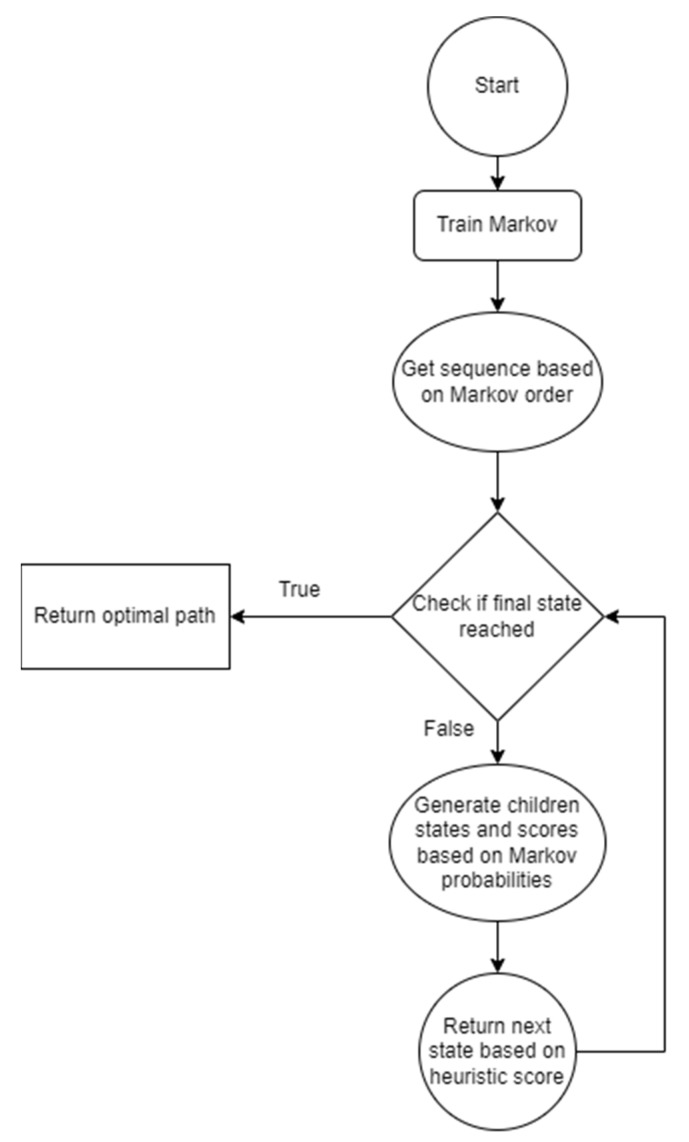
Prediction pipeline and data flow of the algorithm.

The A* algorithm achieves an O(log H(x)) time complexity due to the optimal heuristic and an ${O(b}^{d})$ space complexity, where b is the branching factor and d is the solution depth. An activity diagram of the algorithm can be seen in Figure 5. Once the Markov chain is trained, a sequence depending on the Markov chain order is taken as input. Unless the final state is reached, the next leaf node is taken based on the heuristic score. If the Markov chain does not return a prediction for the component being picked in the next state, such as only returning one of the six possible moves or none, the missing moves have a score of 1, equal to the path cost, while the predicted moves have a score of 1+P(q), where P(q) is the probability of the q-th move being the next one. For example, if the Markov chain does not return any probabilities, all the next possible moves will have a score of 1; therefore, the next move will be the first item in the possible moves array.

By resetting the score of each move to 1 (the path cost) when the Markov chain does not return a score for the respective path, the algorithm makes sure that the new prediction is not overwritten by the previous results. This was a required addition to the algorithm as, in the way the tablet assembly is done, it will decrease the number of predictions available in the next step by one. So, for example, if the battery was assembled at the first step, and the Markov predictor returned the biggest probability for the battery, if the score would not be reset to 1, the algorithm would try to install the battery, given that there are two battery modules. This would reflect a decrease in the accuracy as there is a probability where the Markov chain did not see two consecutive battery installations and would try to predict a new one. Another reason for resetting the score is to give all the next possible paths an equal chance of being picked in the next step and to make sure that after the node expansion, no score of the previous rounds is carried over as the calculation for the exact distance of the optimal result can be done on the fly.

## 4. Experimental Results

For the evaluation of the novel predictor and its comparison to other predictors in the assembly assistance context of the project, the accuracy, coverage, and prediction rate measurements were used to obtain comparable results and determine the best approach to solve the problem. The accuracy is measured as correct predictions out of the predictions made. The coverage is measured as correct predictions out of all the possible predictions, and prediction rate is measured as the number of predictions generated out of all the possible predictions. The simulation environment and the architecture of the algorithm were designed using Python for ease of implementation. Several open-source libraries were used, such as sklearn and numpy. The Markov implementation was done manually in Python. The evaluations were done on an average laptop consisting of an i5-7300HQ processor at 2.5 GHz and 8 GB memory.

Before observing the metrics, several important features of the novel hybrid algorithm were discovered when checking the results. First, a new level of robustness is achieved by the algorithm, which is essential during use inside assembly assistance stations in the Industry 4.0 era. The algorithm has a prediction rate of 100%, meaning that for any input, it will have an output; moreover, even if the Markov chain predictor would not return any results, the hybrid algorithm will still propose a correct assembly sequence due to the node expansion functionality built inside the A*. This is one of the most useful points of the algorithm as it can be used on any number of unseen assembly states from the training stage.

The second important feature observed when gathering the results is the error detection capability and the ability to correct wrong assemblies; this is one of the most sought out features in Industry 4.0. If the start would be a tablet that is wrongly assembled, by using negative scores for the incorrectly assembled pieces, the A* algorithm would very easily navigate its way back to a valid assembly sequence by recommending the removal of the piece until it reaches a valid state, where the Markov chain would be able to predict future valid assembly sequences.

The data composition is based on randomly sampled data from a mix of correct assembly sequences from a 68-student cohort and the 111 manufacturing workers used in [35] that assembled the tablet using the same methodology. A mixed dataset was chosen to avoid the bias generated by the factory workers, who tended to assemble the tablets in sequential order, whereas the students took other approaches. This resulted in an increase in the generalization power of the predictor and avoided over-fitting. The evaluations are done on the mixed dataset, as well as on the “Students” and “Workers” datasets individually.

During the development process, several iterations of the heuristic were implemented, starting from the naïve approach. The iterations and progress can be seen in the charts below. The models always have a 100% prediction rate due to the tree search part of the prediction; therefore, both coverage and accuracy will always be the same. The A* algorithm was combined with Markov models of orders 1 ÷ 7, denoted as M1 ÷ M7.

**Figure 6:**
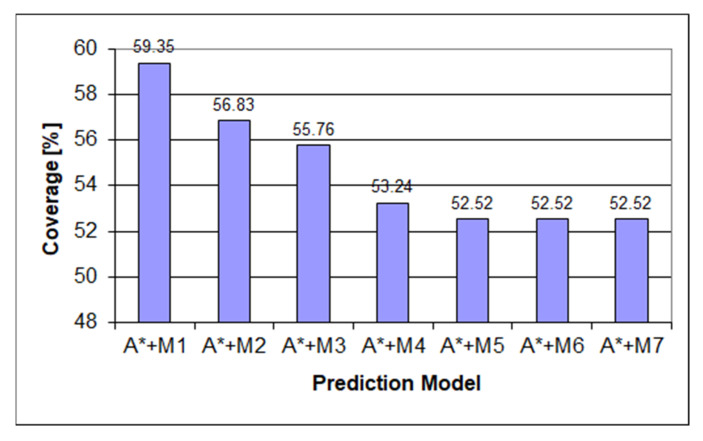
Predictor coverage graph for underestimating heuristic on the mixed dataset.

**Figure 7:**
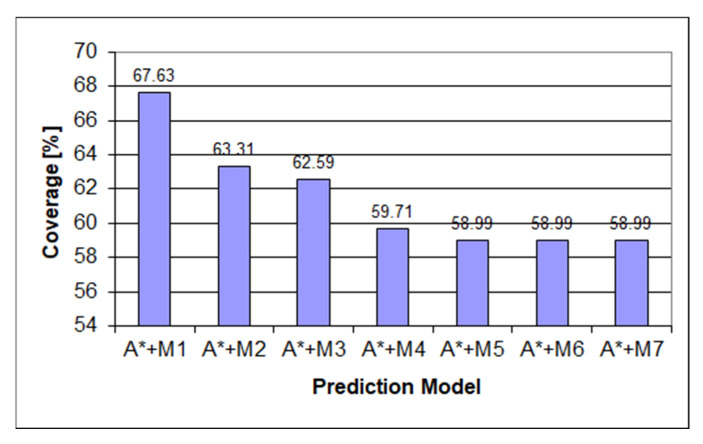
Predictor coverage graph for the perfect heuristic on the mixed dataset.

Figure 6 depicts how the underestimating heuristic underperforms the Markov chain used in the prediction process due to the heuristic score being favored over the Markov prediction score even in cases when the Markov prediction was predicting the correct result. After making the heuristic always return the exact length to the final state, we can see from Figure 7 a considerable increase in the amount of prediction power as the prediction will follow the Markov prediction when available and follow the shortest path score when the Markov prediction is missing. Another part of the heuristic obtaining a considerable score is the ordering of the items in the queue as when no Markov score is present, the last item will be the following child, with the lowest state score described in the methodology used in [28].

**Figure 8:**
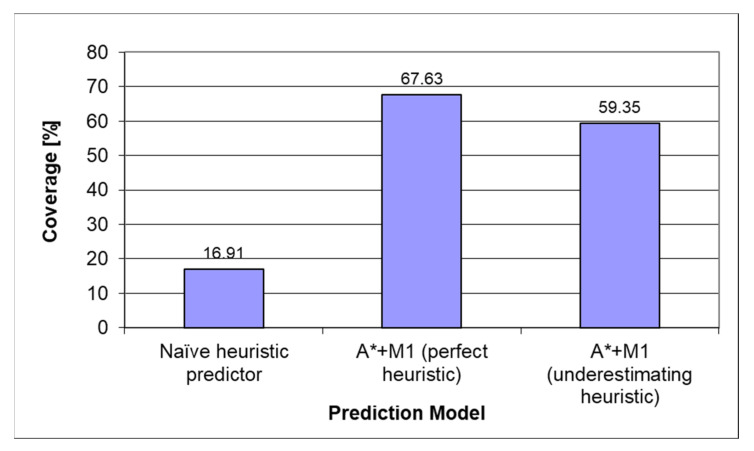
Heuristic comparison on the mixed dataset.

Figure 8 presents a comparison of heuristics on the mixed dataset. The best coverage was provided by the informed tree search algorithm with a first-order Markov model and perfect heuristic.

**Figure 9:**
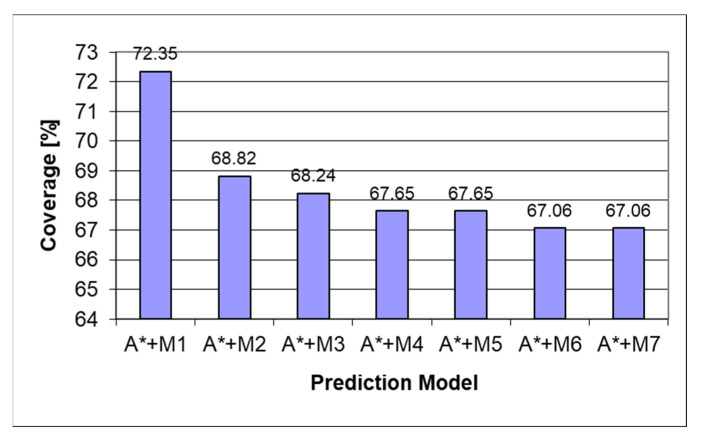
Predictor coverage graph for the perfect heuristic on the “Workers” dataset.

**Figure 10:**
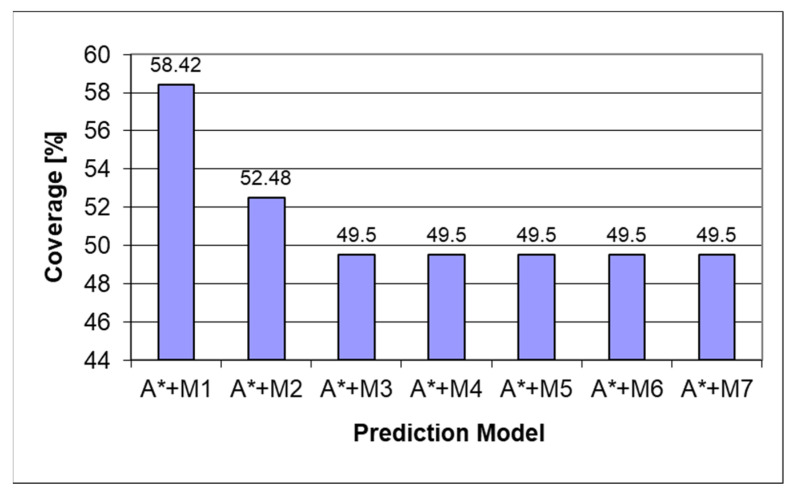
Predictor coverage graph for the perfect heuristic on the “Students” dataset.

Analyzing Figure 9 and Figure 10, similar results to [37] were observed, namely, that for the algorithm, it is easier to learn the behavior of the manufacturing workers, which, based on their experience, are more conservative in their assembly patterns. The increase of 14% in the prediction power can also be attributed to the narrower range of unique assemblies available in the manufacturing dataset compared to the students’ dataset, where several unique assemblies were met; whenever the algorithm predicted equal chances for both of them, the first one added in the queue would be picked.

The results of the best iterations can be found in Table 3, compared with other predictors used in the same context. The base predictor is a naïve heuristic model, where each move has a score of 1, and the assembly prediction is always the same. The novel A* algorithm applied on the top of a first-order Markov chain was also compared with a first-order Markov chain used alone, the PPMN, GBDT, and LSTM models, respectively.

The evaluations show that the proposed A* algorithm, used together with a first-order Markov model, outperforms all the other models in terms of coverage and prediction rate, making it the most suitable algorithm to be used in the assembly station’s prediction process. While the accuracy is comparable between the first-order Markov predictor and the A* with the order 1 Markov model, the main metric that is used for comparison is the coverage that also adds the prediction rate into the metric, rendering a more useful metric for the Industry 4.0 era, where the prediction rate matters.

Another observation can be made in terms of prediction rate, namely that tree-based predictors as well as neural predictors managed to achieve a prediction rate of 100%, which is desirable when implementing the algorithms in real-world uses. This outcome is expected due to the way the algorithms are built, as they will always output a prediction for any input even though the confidence for the prediction is low; therefore, the accuracy is low (see LSTM accuracy metric in Table 3).

The Markovian-based predictors suffer from not having all the scenarios available in the training data, resulting in a lower prediction rate but higher accuracy. This was also observed in a previous study [37], where the feature importance metrics could be observed from the algorithms. The main feature used for prediction was the state of the tablet, with a representation of over 98% of the splits inside the predictor, at least for the GBDT presented in the study. As can be seen in Table 3, the accuracy is similar to that of the Markov-based predictors, with the added benefit of increased coverage due to the tree-based prediction method.

## 5. Conclusions and Further Work

In this paper, a new method based on an informed tree search on top of Markov chain prediction was applied to suggest a plausible move for the next assembly step. The novelty of the method is the hybrid approach that combines an informed tree search with a stochastic model, as well as the usage of this hybrid model for next assembly step prediction. When the Markov chain fails in its prediction due to a new scenario, then an informed tree search is performed. The proposed model is intended to be used as a component of our assembly assistance system, which thus is able to guide factory workers and trainees. The algorithm was configured by choosing the most efficient combination in terms of prediction coverage: an informed tree search with a perfect heuristic relying on the predictions generated by a first-order Markov chain. The comparisons with other existing prediction models (e.g., Markov chains, PPMN, GBDT, LSTM) show that the proposed method provides the best results in terms of coverage, which is the most important evaluation metric as it reflects the percentage of correct assembly step predictions. The coverage of this best model was 67.63%, outperforming by 15.83% the first-order Markov chain, by 5.76% the second-order PPMN, by 2.52% the GBDT, and by 19.43% the LSTM.

There were several limitations present during the study; the main one was that data collection was done in a physical manner by using the assembly station. By collecting data in a physical manner, an increased amount of time was necessary to reset the experiment after each use and for the participants to answer the questionnaire. Another impairment was calibrating the sensors used by the machine as it took several iterations in the calibration process to be able to gather accurate data that could be used for machine learning. Yet another limitation was the COVID-19 pandemic situation, as the experiment involving the factory workers was under pandemic conditions and specific special conditions had to be assured for the safety of the participants. As a scheme limitation, we can mention the necessity of training data.

The novel algorithm can be further improved by replacing the simple heuristic used in the model with a hyper-heuristic that can gain more information from the environment, e.g., instead of assigning a basic score to each component, we can assign a Euclidean score based on how close the component is to the last performed move or a score based on the assembly direction. Another approach would be to calculate the score based on the total remaining available pieces, making the system use alternative assembly pieces when two or more pieces can be used for the same module, thus optimizing the module quantities.

As an outlook, we are targeting the development of a web application to gather more data about assembly sequences to further extend the prediction capacity of the machine learning algorithms. Besides this, we plan to execute further on-site studies with technical and professional school students, considering a broader spectrum of user typologies and basic emotion and mental states as well to cover the requirements from future manufacturing operators. Considering the prediction algorithms, further ones will be investigated, such as dynamic Bayesian networks, hybrid stochastic predictors, and hidden Markov models.

In the longer term, from a software training application perspective, we want to better integrate all the microservices within the training application to provide more robust functionalities by supporting more devices and sensors so that the prototypes move from TRL 3 (i.e., technology validated in the lab) to TRL 5 (i.e., technology validated in the relevant environment), focusing on a real ongoing product manufactured by a regional industrial company.

## Figures and Tables

**Table 1:** Characteristics of Sensytouch ST43 SLIM.

Component	Specifications
Display	43-inch 4K touchscreen
Processor	Intel i7-7700
Graphical Processor	NVIDIA GeForce GTX 1060
Memory	16 GB
Storage	250 GB
Operating System	Windows 10

**Table 2:** Notations.

Notation	Meaning
R	The order of the Markov model
$s_{t}$	The state of the Markov model at time t
H(q)	Heuristic of the distance from state q to the final state
FSS(q)	Final state score of (q)
D(q)	The depth of the current state q
P(q)	The probability of the current state q being the next state

**Table 3:** Comparison between different predictors on the mixed dataset.

Algorithm	Accuracy	Coverage	Prediction Rate
Markov order 1	67.92	51.8	76.26
A* with Markov order 1	67.63	67.63	100
Naïve heuristic predictor	16.91	16.91	100
PPMN order 2	67.19	61.87	92.09
GBDT	65.11	65.11	100
LSTM	48.2	48.2	100

## Data Availability

The data presented in this study are available upon request.
